# Dichloridobis(4-pyridylmethyl 1*H*-pyrrole-2-carboxyl­ate-κ*N*)zinc

**DOI:** 10.1107/S1600536812001353

**Published:** 2012-02-24

**Authors:** Can Li, Guilong Zhang, Zhenming Yin

**Affiliations:** a Tianjin Key Laboratory of Structure and Performance for Functional Molecules, College of Chemistry, Tianjin Normal University, Tianjin 300387, People’s Republic of China; b Agro-Environmental Protection Institute, Ministry of Agriculture, Tianjin 300191, People’s Republic of China

## Abstract

In the title mol­ecule, [ZnCl_2_(C_11_H_10_N_2_O_2_)_2_], the Zn^II^ ion, situated on a twofold axis, is in a distorted tetra­hedral coordination environment formed by two chloride anions and two pyridine N atoms of the two organic ligands. In the pyrrole-2-carboxyl­ate unit, the pyrrole N—H group and the carbonyl group point approximately in the same direction. The dihedral angle between the two pyridine rings is 54.8 (3)°. The complex mol­ecules are connected into chains extending along [101] by N—H⋯Cl hydrogen bonds. The chains are further assembled into (-101) layers by C—H⋯O and C—H⋯Cl inter­actions.

## Related literature

For the hydrogen-bonded assemblies of pyrrole-based structures, see: Wang & Yin (2007 ▶); Yin & Li (2006 ▶); Cui *et al.* (2009 ▶).
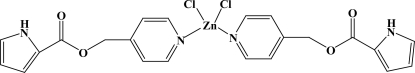


## Experimental

### 

#### Crystal data

[ZnCl_2_(C_11_H_10_N_2_O_2_)_2_]
*M* *_r_* = 540.69
Monoclinic, 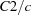

*a* = 27.604 (14) Å
*b* = 6.205 (3) Å
*c* = 16.087 (8) Å
β = 120.309 (6)°
*V* = 2379 (2) Å^3^
*Z* = 4
Mo *K*α radiation
μ = 1.29 mm^−1^
*T* = 296 K
0.26 × 0.20 × 0.14 mm

#### Data collection

Bruker SMART CCD area-detector diffractometer
Absorption correction: multi-scan (*SADABS*; Bruker, 1997 ▶) *T*_min_ = 0.727, *T*_max_ = 1.000
6100 measured reflections
2098 independent reflections
1823 reflections with *I* > 2σ(*I*)
*R*_int_ = 0.018

#### Refinement

*R*[*F* ^2^ > 2σ(*F* ^2^)] = 0.024
*wR*(*F* ^2^) = 0.069
*S* = 1.06
2098 reflections
150 parameters
H-atom parameters constrained
Δρ_max_ = 0.17 e Å^−3^
Δρ_min_ = −0.27 e Å^−3^

### 

Data collection: *SMART* (Bruker, 1997 ▶); cell refinement: *SAINT* (Bruker, 1997 ▶); data reduction: *SAINT*; program(s) used to solve structure: *SHELXS97* (Sheldrick, 2008 ▶); program(s) used to refine structure: *SHELXL97* (Sheldrick, 2008 ▶); molecular graphics: *SHELXTL* (Sheldrick, 2008 ▶); software used to prepare material for publication: *SHELXTL*.

## Supplementary Material

Crystal structure: contains datablock(s) global, I. DOI: 10.1107/S1600536812001353/gk2445sup1.cif

Structure factors: contains datablock(s) I. DOI: 10.1107/S1600536812001353/gk2445Isup2.hkl

Additional supplementary materials:  crystallographic information; 3D view; checkCIF report

## Figures and Tables

**Table 1:** Hydrogen-bond geometry (Å, °)

*D*—H⋯*A*	*D*—H	H⋯*A*	*D*⋯*A*	*D*—H⋯*A*
N2—H2⋯Cl1^i^	0.86	2.57	3.306 (3)	144
C3—H3⋯Cl1^ii^	0.93	2.75	3.495 (3)	138
C4—H4⋯O1^iii^	0.93	2.54	3.354 (3)	146

Symmetry codes: (i) 
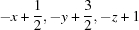
; (ii) 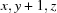; (iii) 
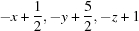
.

**Fig. 1.**
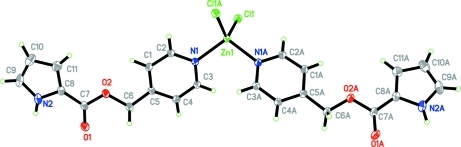
The molecular structure of the title compound with displacement ellipsoids shown at the 30% probability level. The atom with the 'A' label were generated by the symmetry operation -x,y,1/2-z.

**Fig. 2.**
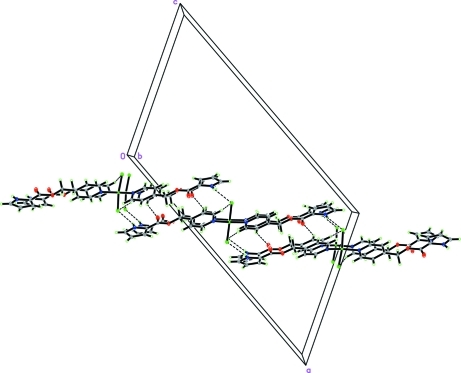
The layer of the title molecules assembled by intermolecular hydrogen bonds (hydrogen bonds are shown as dashed lines).

## supplementary crystallographic information

### Comment

In our earlier works, we have reported the hydrogen-bonded assemblies of 4-pyridylmethyl 1*H*-pyrrole-2-carboxylate (Wang & Yin, 2007) and some other pyrrole-based compounds (Yin & Li, 2006; Cui *et al.* 2009) in the solid state. Combination of coordination bonding and hydrogen bonding is an effective strategy for the generation of supramolecular networks. Continuing our study, herein we report the crystal structure of the complex obtained with 4-pyridylmethyl-1*H*-pyrrole-2-carboxylate and ZnCl_2_.

A perspective view of the title compound with atomic labeling is shown in Fig. 1. The complex consists of one ZnCl_2_ and two ligand molecules, in which both the pyrrole-2-carboxylate moieties adopted *syn* conformation with the carbonyl group arranged in the same direction as the adjacent pyrrole N—H group. In the complex, the dihedral angle between the two pyridine rings is 54.8 (3)°. The complex molecules assemble into layer structrue through N—H···Cl, C—H···O and C—H···Cl hydrogen bonds (Fig. 2).

### Experimental

The methanol solution of ZnCl_2_ (0.1 M, 5 mL) was layered on CHCl_3_ solution of 4-pyridylmethyl 1*H*-pyrrole-2-carboxylate (0.1 M, 10 mL) and then evaporated to give colorless crystals of the title compound in about 70% yield.

### Refinement

All H atoms were placed in calculated positions (C—H = 0.93 -0.97 Å; N-H = 0.86 Å) and refined as riding on their carrier atoms with *U*_iso_(H) = 1.2 *U*_eq_(C, N).

### Crystal data

**Table:**

[ZnCl_2_(C_11_H_10_N_2_O_2_)_2_]	*F*(000) = 1104
*M**_r_* = 540.69	*D*_x_ = 1.510 Mg m^−^^3^
Monoclinic, *C*2/*c*	Melting point: 438 K
Hall symbol: -C 2yc	Mo *K*α radiation, λ = 0.71073 Å
*a* = 27.604 (14) Å	Cell parameters from 2747 reflections
*b* = 6.205 (3) Å	θ = 2.5–26.8°
*c* = 16.087 (8) Å	µ = 1.29 mm^−^^1^
β = 120.309 (6)°	*T* = 296 K
*V* = 2379 (2) Å^3^	Block, colourless
*Z* = 4	0.26 × 0.20 × 0.14 mm

### Data collection

**Table:**

Bruker SMART CCD area-detector diffractometer	2098 independent reflections
Radiation source: fine-focus sealed tube	1823 reflections with *I* > 2σ(*I*)
Graphite monochromator	*R*_int_ = 0.018
phi and ω scans	θ_max_ = 25.0°, θ_min_ = 2.5°
Absorption correction: multi-scan (*SADABS*; Bruker, 1997)	*h* = −32→32
*T*_min_ = 0.727, *T*_max_ = 1.000	*k* = −7→5
6100 measured reflections	*l* = −17→19

### Refinement

**Table:**

Refinement on *F*^2^	Primary atom site location: structure-invariant direct methods
Least-squares matrix: full	Secondary atom site location: difference Fourier map
*R*[*F*^2^ > 2σ(*F*^2^)] = 0.024	Hydrogen site location: inferred from neighbouring sites
*wR*(*F*^2^) = 0.069	H-atom parameters constrained
*S* = 1.06	*w* = 1/[σ^2^(*F*_o_^2^) + (0.0441*P*)^2^ + 0.3857*P*] where *P* = (*F*_o_^2^ + 2*F*_c_^2^)/3
2098 reflections	(Δ/σ)_max_ < 0.001
150 parameters	Δρ_max_ = 0.17 e Å^−^^3^
0 restraints	Δρ_min_ = −0.27 e Å^−^^3^

### Special details

**Table:**

Geometry. All e.s.d.'s (except the e.s.d. in the dihedral angle between two l.s. planes) are estimated using the full covariance matrix. The cell e.s.d.'s are taken into account individually in the estimation of e.s.d.'s in distances, angles and torsion angles; correlations between e.s.d.'s in cell parameters are only used when they are defined by crystal symmetry. An approximate (isotropic) treatment of cell e.s.d.'s is used for estimating e.s.d.'s involving l.s. planes.
Refinement. Refinement of *F*^2^ against ALL reflections. The weighted *R*-factor *wR* and goodness of fit *S* are based on *F*^2^, conventional *R*-factors *R* are based on *F*, with *F* set to zero for negative *F*^2^. The threshold expression of *F*^2^ > σ(*F*^2^) is used only for calculating *R*-factors(gt) *etc*. and is not relevant to the choice of reflections for refinement. *R*-factors based on *F*^2^ are statistically about twice as large as those based on *F*, and *R*- factors based on ALL data will be even larger.

### Fractional atomic coordinates and isotropic or equivalent isotropic displacement parameters (Å^2^)

**Table:**

	*x*	*y*	*z*	*U*_iso_*/*U*_eq_	
Zn1	0.0000	0.41749 (4)	0.2500	0.03878 (12)	
Cl1	0.01743 (2)	0.23365 (8)	0.14847 (3)	0.05019 (15)	
O1	0.30330 (6)	1.1722 (3)	0.65205 (11)	0.0654 (4)	
O2	0.24547 (5)	0.8880 (2)	0.60656 (9)	0.0495 (3)	
N1	0.06598 (6)	0.6110 (2)	0.33725 (10)	0.0395 (3)	
N2	0.37959 (7)	0.9421 (3)	0.81985 (12)	0.0555 (5)	
H2	0.3931	1.0663	0.8187	0.067*	
C1	0.15031 (8)	0.6615 (3)	0.48714 (13)	0.0481 (5)	
H1	0.1754	0.6095	0.5483	0.058*	
C2	0.10377 (8)	0.5440 (3)	0.42633 (14)	0.0470 (5)	
H2A	0.0980	0.4122	0.4475	0.056*	
C3	0.07520 (7)	0.8013 (3)	0.30828 (13)	0.0405 (4)	
H3	0.0494	0.8502	0.2469	0.049*	
C4	0.12083 (8)	0.9263 (3)	0.36502 (14)	0.0427 (4)	
H4	0.1258	1.0569	0.3420	0.051*	
C5	0.15987 (7)	0.8576 (3)	0.45746 (13)	0.0394 (4)	
C6	0.20924 (8)	0.9974 (3)	0.51816 (14)	0.0501 (5)	
H6A	0.2293	1.0285	0.4844	0.060*	
H6B	0.1968	1.1328	0.5313	0.060*	
C7	0.29241 (8)	0.9944 (4)	0.66858 (13)	0.0444 (4)	
C8	0.32739 (8)	0.8683 (3)	0.75342 (13)	0.0460 (4)	
C9	0.40637 (10)	0.7894 (5)	0.88712 (16)	0.0712 (7)	
H9	0.4427	0.7994	0.9392	0.085*	
C10	0.37168 (12)	0.6193 (5)	0.86637 (18)	0.0767 (8)	
H10	0.3796	0.4930	0.9020	0.092*	
C11	0.32176 (10)	0.6672 (4)	0.78171 (16)	0.0607 (5)	
H11	0.2904	0.5784	0.7504	0.073*	

### Atomic displacement parameters (Å^2^)

**Table:**

	*U*^11^	*U*^22^	*U*^33^	*U*^12^	*U*^13^	*U*^23^
Zn1	0.03178 (17)	0.03411 (18)	0.03693 (18)	0.000	0.00734 (13)	0.000
Cl1	0.0473 (3)	0.0494 (3)	0.0450 (3)	0.0092 (2)	0.0167 (2)	−0.0014 (2)
O1	0.0573 (9)	0.0543 (9)	0.0588 (9)	−0.0232 (8)	0.0102 (8)	0.0022 (8)
O2	0.0393 (7)	0.0473 (8)	0.0451 (7)	−0.0124 (6)	0.0089 (6)	0.0012 (6)
N1	0.0343 (8)	0.0366 (8)	0.0373 (8)	−0.0042 (6)	0.0104 (7)	0.0007 (6)
N2	0.0416 (9)	0.0756 (13)	0.0404 (9)	−0.0067 (9)	0.0142 (8)	−0.0053 (8)
C1	0.0428 (10)	0.0460 (11)	0.0378 (10)	−0.0054 (9)	0.0071 (9)	0.0065 (9)
C2	0.0450 (11)	0.0395 (11)	0.0426 (10)	−0.0090 (8)	0.0118 (9)	0.0066 (8)
C3	0.0382 (10)	0.0378 (10)	0.0368 (9)	0.0010 (8)	0.0124 (8)	0.0037 (8)
C4	0.0404 (10)	0.0370 (10)	0.0457 (10)	−0.0039 (8)	0.0181 (9)	0.0047 (8)
C5	0.0341 (9)	0.0381 (9)	0.0416 (10)	−0.0050 (8)	0.0159 (8)	−0.0028 (8)
C6	0.0425 (11)	0.0455 (11)	0.0460 (11)	−0.0106 (9)	0.0103 (9)	0.0023 (9)
C7	0.0357 (10)	0.0500 (11)	0.0417 (10)	−0.0097 (9)	0.0152 (9)	−0.0063 (9)
C8	0.0386 (10)	0.0565 (12)	0.0412 (10)	−0.0040 (9)	0.0190 (9)	−0.0036 (9)
C9	0.0527 (13)	0.112 (2)	0.0409 (12)	0.0165 (15)	0.0176 (11)	0.0113 (13)
C10	0.0786 (18)	0.092 (2)	0.0623 (15)	0.0227 (16)	0.0379 (14)	0.0292 (14)
C11	0.0610 (13)	0.0659 (14)	0.0588 (13)	−0.0012 (12)	0.0328 (12)	0.0075 (11)

### Geometric parameters (Å, º)

**Table:**

Zn1—N1	2.0367 (15)	C2—H2A	0.9300
Zn1—N1^i^	2.0367 (15)	C3—C4	1.364 (3)
Zn1—Cl1^i^	2.2347 (9)	C3—H3	0.9300
Zn1—Cl1	2.2347 (9)	C4—C5	1.392 (3)
O1—C7	1.208 (3)	C4—H4	0.9300
O2—C7	1.344 (2)	C5—C6	1.490 (3)
O2—C6	1.431 (2)	C6—H6A	0.9700
N1—C3	1.340 (2)	C6—H6B	0.9700
N1—C2	1.344 (2)	C7—C8	1.441 (3)
N2—C9	1.345 (3)	C8—C11	1.364 (3)
N2—C8	1.369 (3)	C9—C10	1.349 (4)
N2—H2	0.8600	C9—H9	0.9300
C1—C2	1.368 (3)	C10—C11	1.396 (3)
C1—C5	1.380 (3)	C10—H10	0.9300
C1—H1	0.9300	C11—H11	0.9300
			
N1—Zn1—N1^i^	107.76 (9)	C1—C5—C4	117.34 (17)
N1—Zn1—Cl1^i^	104.16 (6)	C1—C5—C6	123.90 (17)
N1^i^—Zn1—Cl1^i^	110.92 (6)	C4—C5—C6	118.76 (17)
N1—Zn1—Cl1	110.92 (6)	O2—C6—C5	108.94 (16)
N1^i^—Zn1—Cl1	104.16 (6)	O2—C6—H6A	109.9
Cl1^i^—Zn1—Cl1	118.61 (4)	C5—C6—H6A	109.9
C7—O2—C6	115.69 (15)	O2—C6—H6B	109.9
C3—N1—C2	117.51 (15)	C5—C6—H6B	109.9
C3—N1—Zn1	122.71 (12)	H6A—C6—H6B	108.3
C2—N1—Zn1	119.71 (12)	O1—C7—O2	122.62 (18)
C9—N2—C8	109.1 (2)	O1—C7—C8	125.59 (17)
C9—N2—H2	125.4	O2—C7—C8	111.77 (17)
C8—N2—H2	125.4	C11—C8—N2	107.33 (19)
C2—C1—C5	119.81 (18)	C11—C8—C7	132.68 (19)
C2—C1—H1	120.1	N2—C8—C7	119.74 (18)
C5—C1—H1	120.1	N2—C9—C10	108.6 (2)
N1—C2—C1	122.78 (17)	N2—C9—H9	125.7
N1—C2—H2A	118.6	C10—C9—H9	125.7
C1—C2—H2A	118.6	C9—C10—C11	107.6 (2)
N1—C3—C4	122.79 (17)	C9—C10—H10	126.2
N1—C3—H3	118.6	C11—C10—H10	126.2
C4—C3—H3	118.6	C8—C11—C10	107.3 (2)
C3—C4—C5	119.77 (17)	C8—C11—H11	126.3
C3—C4—H4	120.1	C10—C11—H11	126.3
C5—C4—H4	120.1		
			
N1^i^—Zn1—N1—C3	−34.15 (12)	C7—O2—C6—C5	179.31 (16)
Cl1^i^—Zn1—N1—C3	−152.03 (13)	C1—C5—C6—O2	4.0 (3)
Cl1—Zn1—N1—C3	79.27 (15)	C4—C5—C6—O2	−175.71 (16)
N1^i^—Zn1—N1—C2	149.00 (17)	C6—O2—C7—O1	1.0 (3)
Cl1^i^—Zn1—N1—C2	31.12 (15)	C6—O2—C7—C8	−177.45 (16)
Cl1—Zn1—N1—C2	−97.58 (15)	C9—N2—C8—C11	1.1 (2)
C3—N1—C2—C1	0.1 (3)	C9—N2—C8—C7	−173.90 (18)
Zn1—N1—C2—C1	177.12 (16)	O1—C7—C8—C11	−178.6 (2)
C5—C1—C2—N1	−0.1 (3)	O2—C7—C8—C11	−0.2 (3)
C2—N1—C3—C4	0.2 (3)	O1—C7—C8—N2	−5.1 (3)
Zn1—N1—C3—C4	−176.75 (14)	O2—C7—C8—N2	173.28 (17)
N1—C3—C4—C5	−0.5 (3)	C8—N2—C9—C10	−1.4 (3)
C2—C1—C5—C4	−0.2 (3)	N2—C9—C10—C11	1.2 (3)
C2—C1—C5—C6	−179.95 (19)	N2—C8—C11—C10	−0.4 (2)
C3—C4—C5—C1	0.5 (3)	C7—C8—C11—C10	173.7 (2)
C3—C4—C5—C6	−179.77 (18)	C9—C10—C11—C8	−0.5 (3)

Symmetry code: (i) −*x*, *y*, −*z*+1/2.

### Hydrogen-bond geometry (Å, º)

**Table:**

*D*—H···*A*	*D*—H	H···*A*	*D*···*A*	*D*—H···*A*
N2—H2···Cl1^ii^	0.86	2.57	3.306 (3)	144
C3—H3···Cl1^iii^	0.93	2.75	3.495 (3)	138
C4—H4···O1^iv^	0.93	2.54	3.354 (3)	146

Symmetry codes: (ii) −*x*+1/2, −*y*+3/2, −*z*+1; (iii) *x*, *y*+1, *z*; (iv) −*x*+1/2, −*y*+5/2, −*z*+1.
